# MicroRNA-330-3p promotes brain metastasis and epithelial-mesenchymal transition via GRIA3 in non-small cell lung cancer

**DOI:** 10.18632/aging.102201

**Published:** 2019-09-08

**Authors:** Chunhua Wei, Ruiguang Zhang, Qian Cai, Xican Gao, Fan Tong, Jihua Dong, Yu Hu, Gang Wu, Xiaorong Dong

**Affiliations:** 1 Cancer Center, Union Hospital, Tongji Medical College, Huazhong University of Science and Technology, Wuhan 430022, China; 2 Medical Research Center, Union Hospital, Tongji Medical College, Huazhong University of Science and Technology, Wuhan 430022, China; 3 Institute of Hematology, Union Hospital, Tongji Medical College, Huazhong University of Science and Technology, Wuhan 430022, China

**Keywords:** non-small cell lung cancer, brain metastasis, miR-330-3p, GRIA3, TGF-β1

## Abstract

Brain metastasis (BM) is associated with poor prognosis in patients with non-small cell lung cancer (NSCLC). We sought to identify microRNAs (miRNAs) that could serve as biomarkers to differentiate NSCLC patients with and without BM. Logistic regression was conducted with 122 NSCLC patients (60 without BM, 62 with BM) to assess the association between miRNAs and BM. We confirmed several risk factors for BM and revealed that serum miR-330-3p levels are higher in NSCLC patients with BM than that without BM. Overexpression of miR-330-3p promoted proliferation, migration, invasion and epithelial-mesenchymal transition (EMT) of NSCLC cells *in vitro* and NSCLC tumorigenesis *in vivo*. Knocking down miR-330-3p suppressed this metastatic phenotype. We identified putative miR-330-3p target genes by comparing mRNA microarray analysis data from A549 cells after miR-330-3p knockdown with candidate miR-330-3p target genes predicted by public bioinformatic tools and luciferase reporter assays. We found that GRIA3 is a target of miR-330-3p and that miR-330-3p stimulates EMT progress by mediating GRIA3-TGF-β1 interaction. Our results provide novel insight into the role of miR-330-3p in NSCLC metastasis, and suggest miR-330-3p may be a useful biomarker for identifying NSCLC with metastatic potential.

## INTRODUCTION

Brain metastasis (BM) is the leading cause of poor prognosis, recurrence, and death in patients with non-small-cell lung cancer (NSCLC). In nearly 10% of NSCLC patients, BM is already present at diagnosis [1], and develops eventually in 40-55% of these patients [2]. The median overall survival (OS) of NSCLC patients with BM is only 4–6 months after whole brain radiotherapy (WBRT) plus palliative treatment [3]. In patients with limited-stage small-cell lung cancer prophylactic cranial irradiation (PCI) may be of some benefit [4, 5]; however, the potential effects of PCI in NSCLC remain controversial [6, 7]. As a result, it is important to identify the risk factors for BM in patients with NSCLC.

MicroRNAs (miRNAs) are a class of conserved small non-coding RNAs 18-25 nucleotides in length [8–10]. A variety of miRNAs have been shown to possess oncogenic or tumor suppressor activity [11]. Generally, a single miRNA regulates hundreds of genes [12, 13]. Thus, miRNA profiling is an efficient alternative to gene expression profiling.

Several miRNAs are known to play roles in the development of NSCLC [14]. In preclinical studies, for example, NSCLC metastasis could be inhibited through manipulation of these miRNAs [15–17]. In studies of human patients, NSCLC BM was associated with miRNA-328 and miRNA-378 [14, 18]. Moreover, recent studies suggest that miR-330-3p contributes to oncogenesis in glioblastoma, colorectal cancer, and esophageal cancer [19–21]. Elevated miR-330-3p expression promotes the proliferation, survival, migration, and invasion of human esophageal cancer cells *in vitro*, and tumor formation in nude mice [21]. In our previous study, we demonstrated that miR-330-3p promote NSCLC invasion and metastasis [22]. In the present study, we investigated the potential role of miR-330-3p in NSCLC BM. Our findings indicate that serum and tissue miR-330-3p levels are higher in NSCLC patients with BM than those without BM, and that miR-330-3p may be a useful biomarker for identifying NSCLC with metastatic potential.

## RESULTS

### Human sample characteristics

The study included a total of 122 NSCLC patients, 62 with BM (BM+), and the remaining 60 without BM (BM-) upon diagnosis (Supplementary Table 1). The two groups did not differ in age. The female sex was overrepresented in the BM+ group (49/62 *vs.* 23/60). Tumor types in the BM+ group included adenocarcinoma (*n* = 54) and squamous cell carcinoma (*n* = 8). Lymph node was involved in 58/62 subjects. EGFR mutations included exon 19 deletions (*n* = 22) and L858R substitution in exon 21 (*n* = 20). The BM- group included adenocarcinoma (*n* = 41), squamous cell carcinomas (*n* = 10), carcinosarcomas (*n* = 2), large cell carcinoma (*n* = 1) and neuroendocrine carcinomas (*n* = 6). Lymph node metastasis was present in 51/60 subjects. EGFR mutation included exon 19 (*n* = 17) and exon 21 (*n* = 15).

Univariate analysis revealed significant associations of BM with the female gender, young age < 60 years, adenocarcinoma type, N2 or N3 lymph node metastasis, *EGFR*19 exon mutation and miR-328, miR-330-3p, miR-350 expression (*P* < 0.05, Supplementary Table 2). Multivariate logistic regression analysis revealed the following predictors of BM: female gender, age < 60 years, adenocarcinoma type, N2 or N3, *EGFR*19 exon mutation and miR-328, miR-330-3p expression (*P* < 0.05, Supplementary Table 2).

### MiR-330-3p distinguished BM+ from BM- patients and predicted BM occurrence

**Figure 1:**
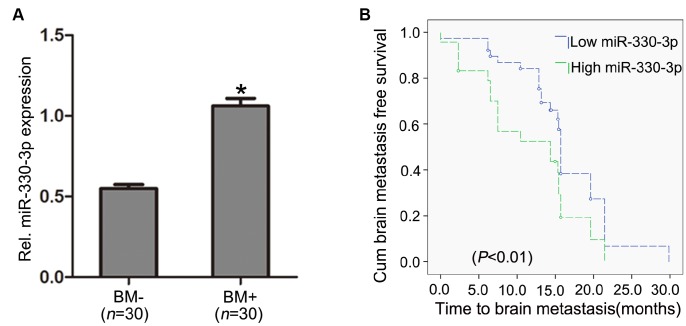
**MiR-330-3p expression in primary lung tissues.** (**A**) miR-330-3p expression was upregulated in primary lung tumor tissues with BM (BM+) compared with subjects without BM (BM-) upon diagnosis (n = 30 each). (**B**) Kaplan-Meier analysis of association between miRNA-330-3p and BM- free period.

Serum miR-328 (*P* = 0.05) and miR-330-3p (*P* = 0.02) were significantly higher in BM+ patients, whereas miR-325, miR-326, miR-370 and miR-500-5p did not differ between the BM+ and BM- groups (Supplementary Table 3). Quantitative real-time PCR revealed higher miR-330-3p in the primary lung lesions in subjects with BM than in subjects without BM upon diagnosis (*n* = 30 each, *P =* 0.003, Figure 1A). Among the 60 patients with no BM upon diagnosis, 23 developed BM during the follow-up period (the median follow-up time was 17 months); the percentage of the patients who developed BM was higher in patients with high (above sample median) circulating miR-330-3p than subjects with low circulating miR-330-3p (*P* = 0.02). Kaplan-Meier analysis revealed shorter time to BM development with higher miR-330-3p (*P* < 0.01, Figure 1B).

### MiR-330-3p promoted proliferation, suppressed apoptosis and facilitated G1-S transition of NSCLC cells

We firstly explored the effects of miR-330-3p on NSCLC cells progress. Our previous work had demonstrated that the expression of miR-330-3p in NSCLC cell lines (A549, H460, HCC827, H1975 and PC-9) was significantly higher than in normal human bronchial epithelial cell line (BEAS-2B) [22]. In this study, we selected A549 (wild-type EGFR) and HCC827 (EGFR mutation at exon 19) cells as representative NSCLC cells.

For each cell line (A549 or HCC827), 3 types of stably transfected cells were generated: cells transfected with empty lentivirus, cells transfected with lentivirus overexpressing miR-330-3p, and cells transfected with anti-miR-330-3p lentivirus. Cells not subjected to viral transfection were included in experiments as an additional control. Transfection was verified using immunofluorescence staining (Supplementary Figure 1A) and qRT-PCR (Supplementary Figure 1B).

**Figure 2:**
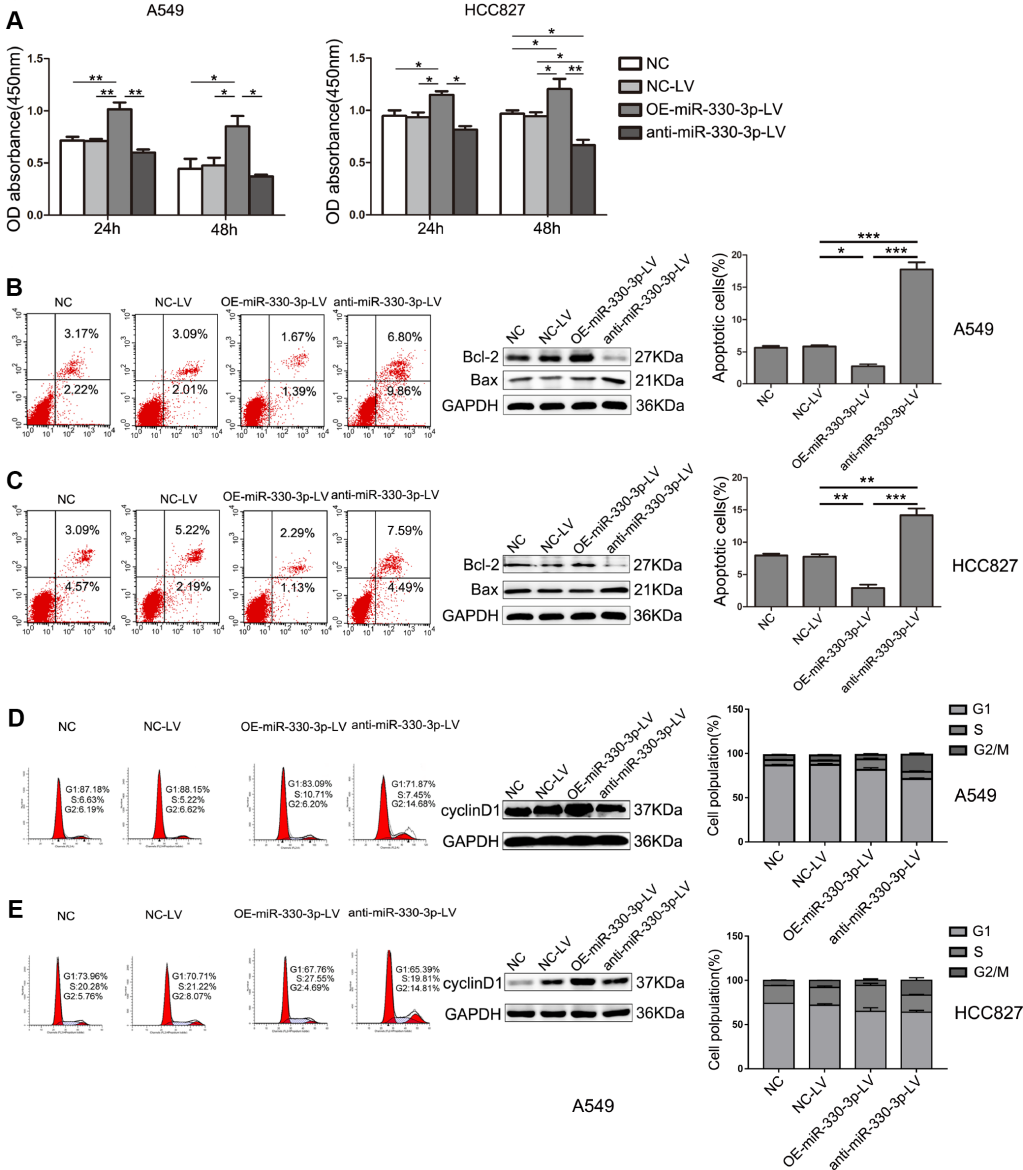
**MiR-330-3p regulated proliferation, apoptosis and cell cycle of NSCLC cells.** (**A**) The proliferative ability of A549 and HCC827cells after transfection was evaluated by MTT assay. Data represent mean ± SD. (**B**, **C**) The apoptosis of A549 and HCC827 cells was determined by Annexin V-fluorescein isothiocyanate (FITC)/7-amino-actinomycin D (7-AAD) staining. The percentages of Annexin-V-positive cells were indicated. The expression of Bax and Bcl-2 was determined by western blotting in A549 and HCC827 cells. GAPDH was used as a loading control. (**D**, **E**) The cell cycle was analyzed by flow cytometry after PI staining, and the data were processed with ModFit LT program. Western blotting of cyclin D1 was shown under each band. Protein level quantification was normalized to GAPDH. *P < 0.05, **P < 0.01, ***P < 0.001.

Proliferation was significantly increased by overexpressing miR-330-3p in both A549 and HCC827 cells at 24h and 48h, and decreased by miR-330-3p knockdown in HCC827 cells at 48h (*P* < 0.05, Figure 2A). Transfection with lentivirus alone did not affect cell proliferation.

Flow cytometry showed that apoptosis was inhibited in overexpressing miR-330-3p-LV in both A549 cells and HCC827 cells compared with NC-LV groups (3.06% *vs.* 5.10%, 3.42% *vs*. 7.41%, respectively, Figure 2B, 2C). In contrast, cell apoptosis was increased by anti-miR-330-3p in A549 cells and HCC827 cells (16.66% *vs.* 5.10%, 12.08% *vs.* 7.41%, respectively; Figure 2B, 2C). Furthermore, the protein expression levels of Bax, Bcl-2, Bak, cleaved PARP and cleaved caspase 3, which are important apoptosis-associated proteins, were detected by western blotting. As shown, overexpressing miR-330-3p increased Bcl-2 and reduced Bax, Bak, cleaved PARP and cleaved caspase 3 expression (Figure 2B, 2C and Supplementary Figure 2A).

The effect of miR-330-3p on the cell cycle distribution was determined using flow cytometry. The results showed that overexpressing miR-330-3p increased the percentage of cells in the S phase, and decreased the percentage of cells in the G1 phase in both A549 and HCC827 cells (Figure 2D, 2E). To further elucidate the specific regulatory proteins responsible for the cell cycle arrest, we explored the effect of miR-330-3p on the cell cycle regulatory proteins cyclin D1, CDK4, CDK6, p21 and p27. As demonstrated, overexpressing miR-330-3p enhanced cyclin D1, CDK4 and CDK6 expression, decreased p21 and p27 expression (Figure 2D, 2E and Supplementary Figure 2B).

### MiR-330-3p promoted NSCLC cells migration, invasion and angiogenesis *in vitro*

**Figure 3:**
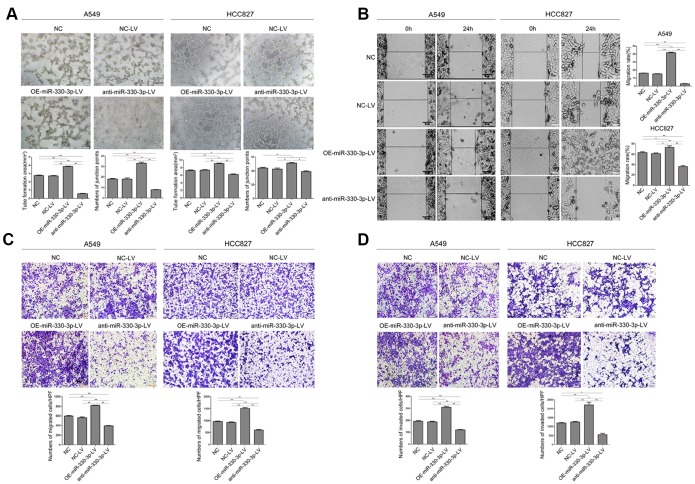
**MiR-330-3p promoted tube formation, cell migration and invasion in A549 and HCC827 cells.** (**A**) Tube formation assay measuring proangiogenic activity in A549 and HCC827 cells; tube formation was assessed using an inverted light microscope (Olympus IX71, original magnification ×100). (**B**) Wound-healing assays were performed to assess NSCLC cell migration. Wound closure was determined 24 h after the scratch. (**C**) Transwell migration assay measuring NSCLC cell migration in A549 and HCC827 cells stably transfected with NC-LV, OE-miR-330-3p-LV or anti-miR-330-3p-LV, respectively. (**D**) Transwell invasion assay was used to quantify cell invasion in a Matrigel-coated chamber. The average number of cells migrating per field of view in three different experiments is plotted. *P < 0.05, **P < 0.01.

Overexpressing miR-330-3p increased the capillary-forming ability in both A549 and HCC827 cells (Figure 3A). In cells transfected with the anti-miR-330-3p-LV, the tubular structure was incomplete and fluffy. The wound-healing assay showed increased migration by overexpressing miR-330-3p and decreased migration by the anti-miR-330-3p (Figure 3B). Transwell migration and invasion assay showed similar results (Figure 3C, 3D).

**Figure 4:**
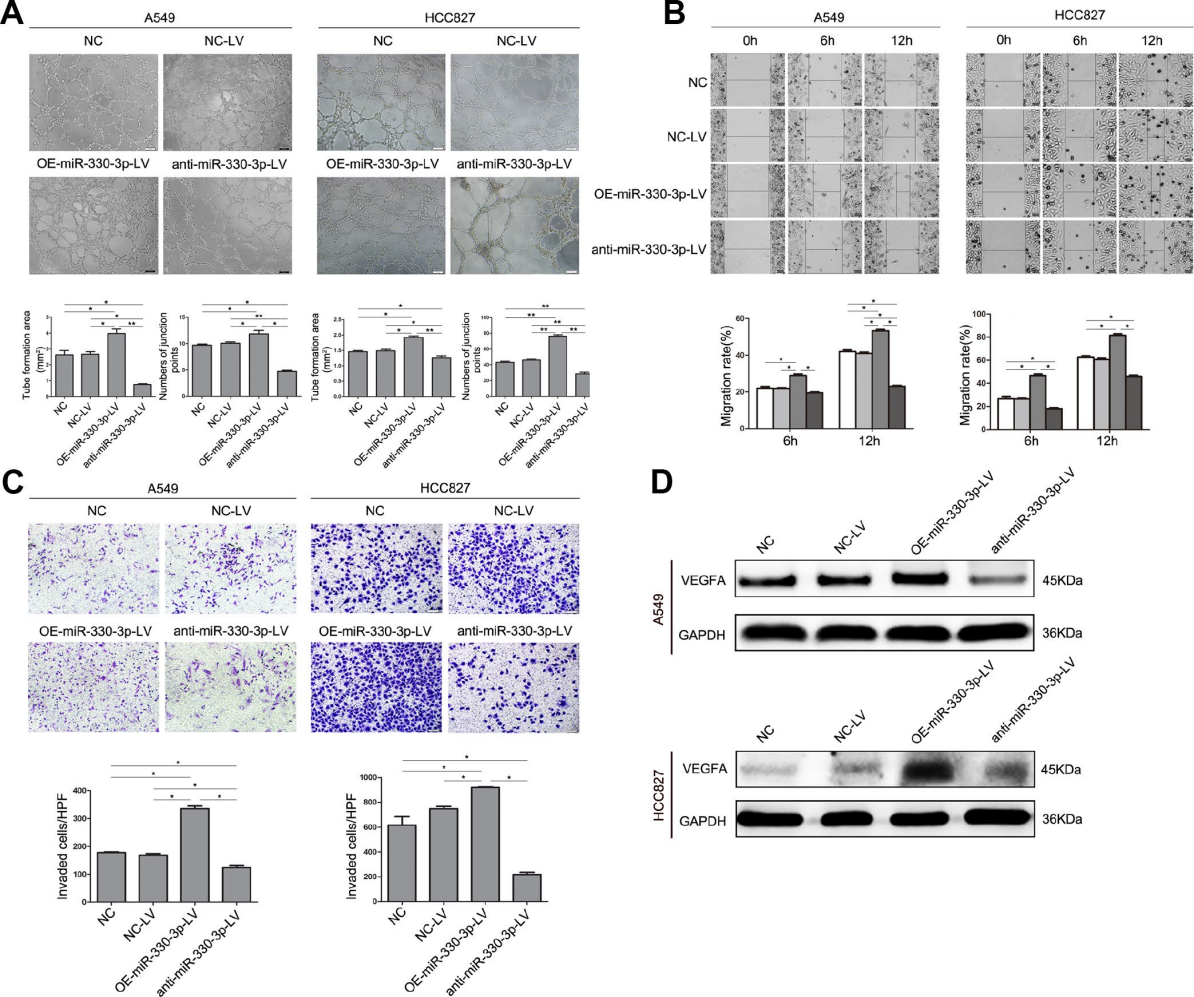
**MiR-330-3p promoted angiogenesis, cell migration and invasion of HUVEC cells co-cultured with A549 and HCC827 cells.** (**A**) HUVECs were seeded on top of extracellular matrix, in the presence of conditioned medium (CM) obtained from NC-LV, OE-miR-330-3p-LV or anti-miR-330-3p-LV-transfected A549 and HCC827 cells. Tube formation was assessed using an inverted light microscope (original magnification ×100). (**B**) HUVECs were seeded in 6-well plates, in the presence of CM obtained from NC-LV, OE-miR-330-3p-LV or anti-miR-330-3p-LV transfected A549 and HCC827 cells. Wound closure was determined 6 h and 12 h after the scratch. (**C**) Cell invasion was evaluated using a 24-transwell chamber with 8-μm pore insert. HUVECs were seeded in the upper chamber, in the presence of CM obtained from NC-LV, OE-miR-330-3p-LV or anti-miR-330-3p-LV-transfected A549 and HCC827 cells. The migrated cells were crystal violet-stained and assessed using an inverted light microscope (×100). (**D**) The expression of VEGFA by western blotting. *P < 0.05, **P < 0.01.

Experiments in HUVEC cells cultured with conditioned medium from A549 or HCC827 cells showed that tube formation, migration and invasion of HUVEC cells were enhanced by overexpressing miR-330-3p in NSCLC cells and decreased by anti-miR-330-3p (*P* < 0.05 for both, Figure 4A–4C). Moreover, VEGF family was considered plays a crucial role in tumor angiogenesis, and VEGFA mediates the leading role. We detected the expression levels of VEGF family, and found that overexpressing miR-330-3p elevated the level of VEGFA and PIGF expression, knockdown of miR-330-3p inhibited the expression of VEGFA and VEGFC (Figure 4D and Supplementary Figure 3).

### MiR-330-3p promoted tumorigenesis and angiogenesis *in vivo*

**Figure 5:**
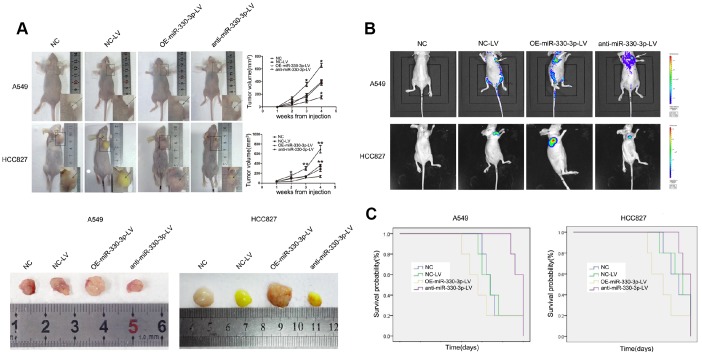
**MiR-330-3p induces tumorigenesis *in vivo*.** (**A**) A549 and HCC827 cells stably transfected with NC-LV, OE-miR-330-3p-LV or anti-miR-330-3p-LV were injected subcutaneously into nude mice. Four weeks after the injection, mice were photographed and killed. Tumor growth curves were obtained. (**B**) Representative bioluminescence images of tumor burden in the mice that received subcutaneous injection of indicated cells. (**C**) Survival curves represent the duration from injection until four weeks when mice were killed. *P < 0.05, **P < 0.01.

**Figure 6:**
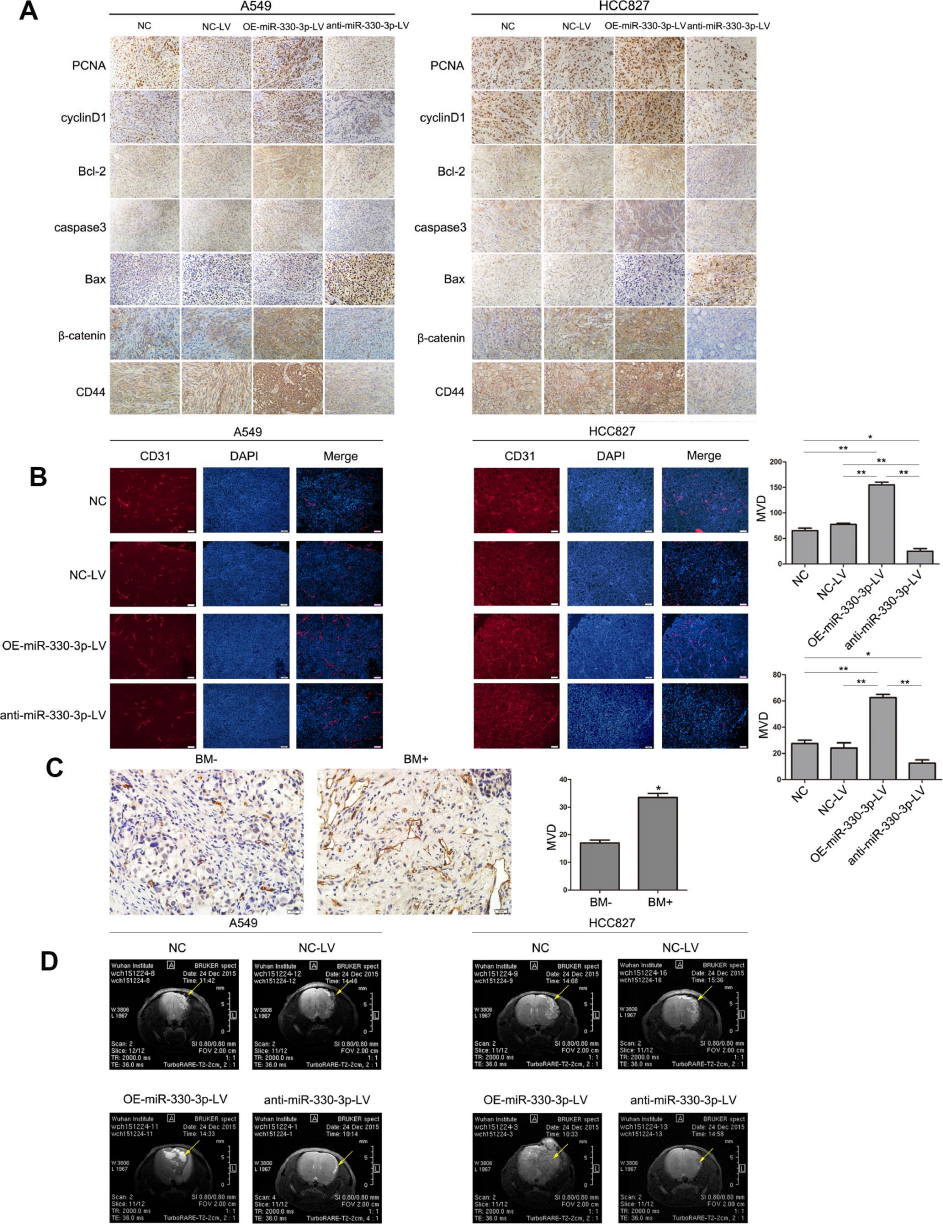
**MiR-330-3p induces tumor metastasis and angiogenesis in BM model.** (**A**) Immunohistochemical analysis of PCNA, cylinD1, Bcl-2, caspase3, Bax, CD44 and β-catenin expression in tissue sections of GFP-labeled tumors isolated from mice injected with A549 and HCC827 cells transfected with NC-LV, OE-miR-330-3p-LV or anti-miR-330-3p-LV. (**B**, **C**) Representative images of CD31-positive endothelial cells in tumor tissues of mice (**B**) and NSCLC patients (**C**). *P < 0.05, **P < 0.01, values are mean ± SD, one-way ANOVA test. (**D**) MRI images of metastatic tumors in the brain. Representative MRI of tumors is shown. A representative experiment of three was reported.

In nude mice receiving subcutaneous injection of transfected A549 or HCC827 cells, implanted tumor grew at a faster rate in mice receiving cells overexpressing miR-330-3p (382.92 ± 89.60 *vs.* 206.68 ± 33.07 mm^3^ in mice receiving empty lentivirus control and *vs.* 212.12 ± 25.45 mm^3^ in mice receiving un-manipulated cells at 4 weeks), and at a slower rate in mice receiving cells with miR-330-3p knockdown (88.40 ± 23.35 mm^3^ at 4 weeks, *P* < 0.05; Figure 5A). The bioluminescence intensity was also enhanced in tumors injected with OE-miR-330-3p-LV NSCLC cells (Figure 5B). The survival time was shorter in mice injected with A549 or HCC827 overexpressing miR-330-3p compared with NC-LV group (*P* < 0.05, Figure 5C). Immunohistochemical analysis revealed that overexpressing miR-330-3p increased PCNA, cyclin D1, CD44 and β-catenin, and reduced cleaved caspase-3 and Bax in tumor tissues (Figure 6A). Immunofluorescence staining revealed decreased CD31 expression and lower number of stained vessels by anti-miR-330-3p (Figure 6B), and decreased CD31 expression in tissues of NSCLC BM- patients compared with BM+ (*P* < 0.05, Figure 6C).

### MiR-330-3p promoted brain metastasis *in vivo*

In nude mice receiving orthotopic implantation directly into the brain, general conditions (weight loss and cancerous cachexia) deteriorated much faster in mice receiving HCC827 or A549 cells overexpressing miR-330-3p. MRI imaging at 25 days demonstrated multiple metastatic foci in mice receiving NSCLC cells overexpressing miR-330-3p *vs.* fewer/smaller orthotopic tumors in mice receiving cells transfected with empty lentivirus or un-manipulated cells (Figure 6D). Most mice receiving A549 and HCC827 cells expressing anti-miR-330-3p did not develop tumor foci (Figure 6D). These results suggested that miR-330-3p promoted the growth of metastatic tumors.

### GRIA3 is a direct target of miR-330-3p

**Figure 7:**
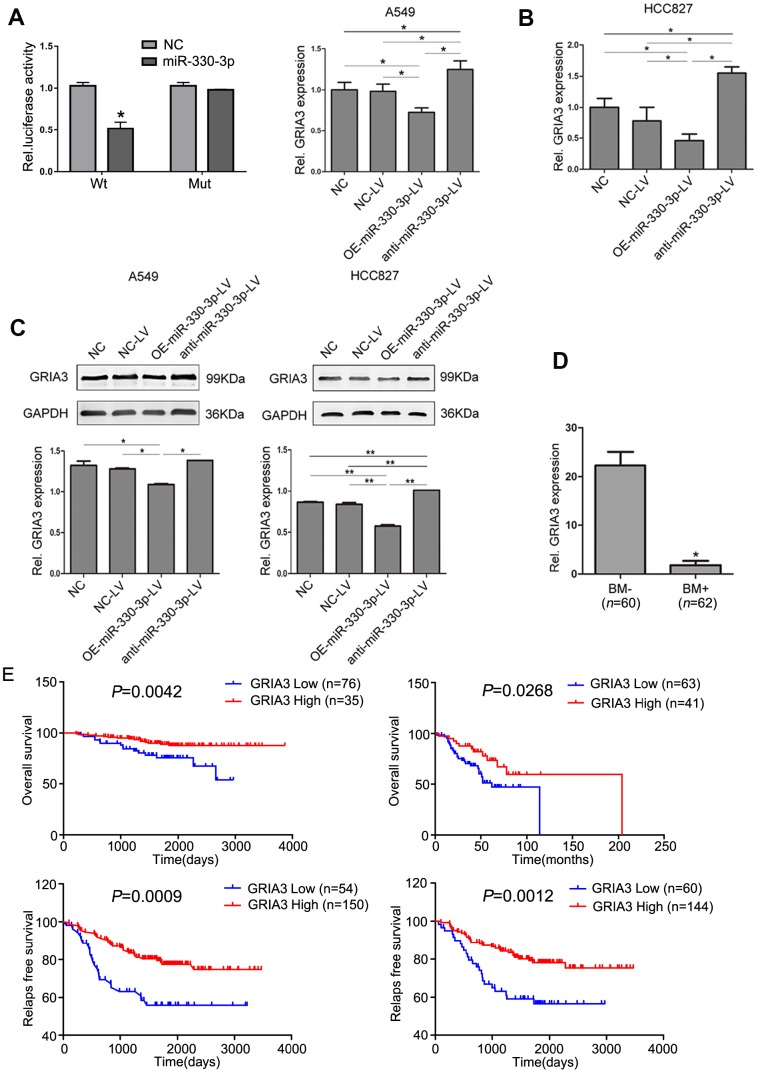
**GRIA3 is a direct target of miR-330-3p.** (**A**) Relative luciferase activity of 293T cells after co-transfection with wild type (Wt) or mutant (Mt) GRIA3 3′ UTR reporter genes and miR-330-3p mimics or control. (**B**) Expression of GRIA3 mRNA in A549 and HCC827-transfected cells was determined by qRT-PCR. (**C**) The expression of GRIA3 was analyzed in A549 and HCC827 transfected cells by western blotting. (**D**) Expression of GRIA3 mRNA in peripheral blood from 62 newly diagnosed BM+ and 60 newly diagnosed BM- NSCLC patients using qRT-PCR. (**E**) Kaplan-Meier plots with log rank test for overall survival and relapse-free survival of lung adenocarcinoma cancer patients with high GRIA3 expression and low GRIA3 expression, respectively. Data represent mean ± SD, *P < 0.05, **P < 0.01. *P* value was calculated by one-way ANOVA.

Bioinformatics analysis using TargetScan, PicTar, HOCTar, Tarbase, miRanda and microCosm identified 4 genes: BMI-1, GRIA3, SOSTDC1 and AGTR2 (Supplementary Table 4). By comparing the candidate miR-330-3p target genes predicted by bioinformatics tools with the candidate target genes identified by the mRNA microarray analysis of A549 cells knocking down miR-330-3p and cells transfected with empty lentivirus (Supplementary Figure 4), we focused on GRIA3 in this study. To determine whether miR-330-3p can directly target its binding sites in the GRIA3 3′-UTR, we generated luciferase reporter vectors containing the wild-type (Wt) or mutant (Mut) sequences of the GRIA3 3’-UTR. The reporter vectors were co-transfected with a miR-330-3p mimic into A549 cells, and the luciferase activity of the Wt reporter gene was inhibited, while not affected in Mut reporter gene (*P* < 0.05, Figure 7A). The expression of GRIA3 in A549 or HCC827 cells was increased by overexpressing miR-330-3p compared with NC-LV groups (*P* = 0.01 and *P* = 0.008, respectively), and decreased by anti-miR-330-3p (*P* = 0.009 and *P* = 0.006, respectively, Figure 7B). Western blotting yielded highly similar results (Figure 7C).

We then evaluated the expression level of GRIA3 in patient samples and found that GRIA3 expression was significantly lower in the 62 NSCLC patients with BM than in those without BM in the serum (*P*< 0.05, Figure 7D). Furthermore, we searched the PrognoScan dataset to determine the association of GRIA3 expression with prognosis. We found that overall survival and relapse free survival was significantly shorter in lung adenocarcinoma patients with low GRIA3 expression than in their counterparts with high GRIA3 expression (Figure 7E).

### Effects of GRIA3 overexpression on NSCLC cells

**Figure 8:**
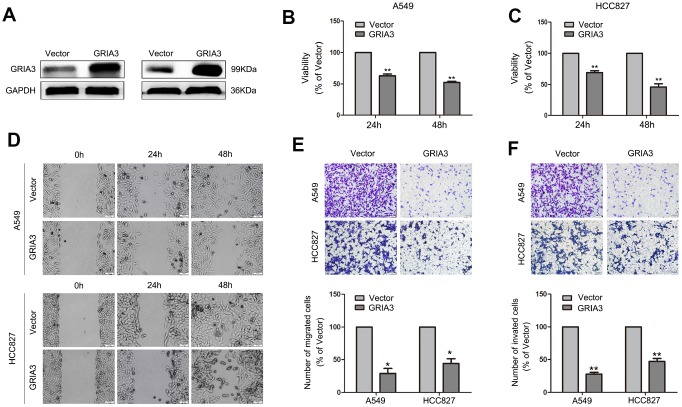
**Effects of GRIA3 overexpression on NSCLC cell viability, migration and invasion *in vitro*.** (**A**) GRIA3 protein expression by western blotting after transfection with GRIA3 or vector. (**B**) Representative images of the MTT assay of A549 and HCC827 cells transfected with GRIA3 or vector. (**C**) Representative images of the wound healing assay of A549 and HCC827 cells transfected with GRIA3 or vector. (**D**, **E**) Representative images (upper) and quantification (lower) of the Transwell migration assay (**D**) and Transwell invasion assay (E) of A549 and HCC827 cells transfected with GRIA3 or vector. Values are mean ± SD, *P < 0.05, **P < 0.01, one-way ANOVA.

In the next experiment, we overexpressed GRIA3 in A549 and HCC827 cells with a pEnter plasmid containing a sequence encoding GRIA3 (Figure 8A). The results showed that overexpression of GRIA3 decreased cell viability in both cell lines (Figure 8B, 8C). Overexpression of GRIA3 in A549 and HCC827 cells decreased the migration and invasiveness (*P* < 0.01, Figure 8D–8F). Furthermore, the zymography assay were performed to evaluate the activity of MMP2 and MMP9. As shown in Supplementary Figure 5, the activity of MMP2 and MMP9 was significantly suppressed by GRIA3 overexpression in A549 and HCC827 cells.

### MiR-330-3p augmented NSCLC cells migration and invasion via TGF-β-induced EMT process

**Figure 9:**
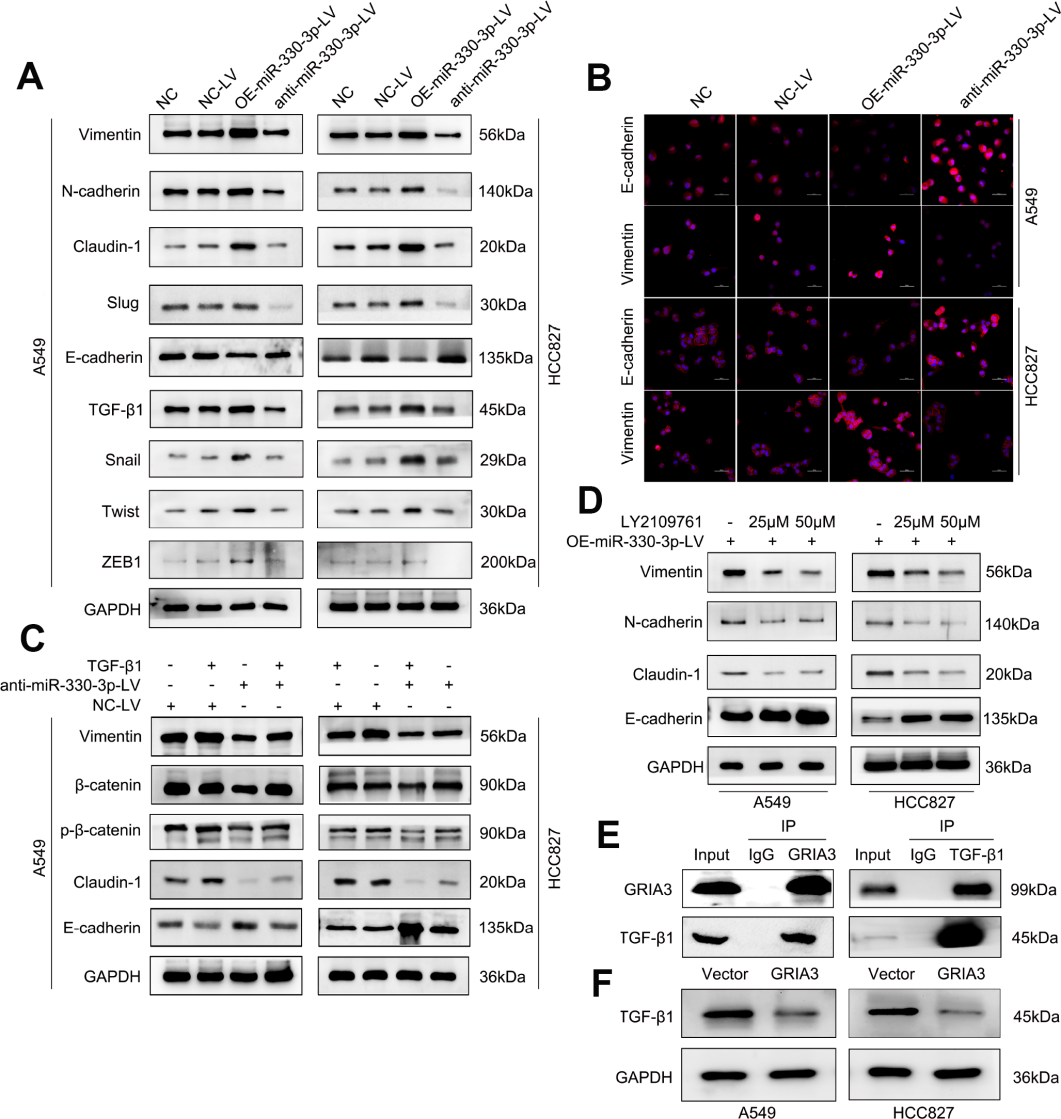
**MiR-330-3p promoted NSCLC cells migration and invasion via TGF-β-induced EMT process.** (**A**) Western blotting analysis of EMT related protein levels in the indicated cells. miR-330-3p could increase mesenchymal phenotypic biomarkers expression and decrease E-cadherin expression. (**B**) Immunofluorescence staining detected the Vimentin and E-cadherin expression in A549 and HCC827 cells. (**C**, **D**) Western blotting analysis of EMT associated protein expression in A549 and HCC827 cells treated with recombinant TGF-β1 (**C**) and the TGF-β1 inhibitor LY2109761 (**D**), respectively. (**E**) Co-IP and Western blotting indicating the endogenous interaction between GRIA3 and TGF-β1 protein in HCC827 cells. (**F**) Western blotting analysis evaluated TGF-β1 expression in A549 and HCC827 cells overexpressing GRIA3.

EMT is a driver of tumor metastasis and is involved in the invasion and migration of tumor cells [23, 24]. Therefore, we evaluated the effects of miR-330-3p on the expression of EMT-related proteins in NSCLC cells. We found that NSCLC cells with miR-330-3p overexpression showed relatively high expression of vimentin, N-cadherin, Slug, Claudin-1, Snail, Twist and ZEB1 (mesenchymal phenotypic biomarkers), and relatively low expression of E-cadherin (an epithelial phenotypic biomarker). In contrast, miR-330-3p knockdown in A549 and HCC827 cells decreased mesenchymal phenotypic biomarkers expression but increased E-cadherin expression (Figure 9A, 9B and Supplementary Figure 6).

It is well known that TGF-β signaling pathway plays a central role in EMT induction and metastasis promotion in cancer [25, 26]. We speculated that miR-330-3p may contribute to TGF-β-mediated EMT in NSCLC cells. Firstly, miR-330-3p overexpression increased TGF-β1 expression (Figure 9A). As shown, treatment of TGF-β1 in A549 and HCC827 cells significantly altered the levels of EMT markers, showing downregulation of E-cadherin and upregulation of β-catenin, Vimentin and Claudin-1. In addition, TGF-β1 reversed the levels of the EMT markers attenuated by knockdown of miR-330-3p in A549 and HCC827 cells (Figure 9C). Furthermore, when treated with LY2109761 (a TGF-β inhibitor) in A549 and HCC827 cells overexpressing miR-330-3p, the simulative effect of miR-330-3p overexpression on EMT was suppressed (Figure 9D).

Next, we endeavored to determine how miR-330-3p regulates TGF-β1. We searched the R2 database to determine any association between GRIA3 and TGF-β1 in lung cancer patient populations. Interestingly, we found a dataset showed that GRIA3 was negatively correlated with TGF-β1 in a set of lung cancer patients (R = -0.455, *P* = 0.0053, Supplementary Figure 7). Furthermore, we searched the Multi-Experiment-Matrix database and found that GRIA3 and TGF-β1 were co-expressed (https://biit.cs.ut.ee/mem/index.cgi). To validate these findings, we performed coimmunoprecipitation assays and demonstrated that GRIA3 physically interacted with TGF-β1 in HCC827 cells (Figure 9E). We further examined the TGF-β1 expression in A549 and HCC827 cells overexpressing GRIA3, and found that the expression of TGF-β1 was decreased in these cells overexpressing GRIA3, showing TGF-β1 was the downstream of GRIA3 (Figure 9F). Taken together, we speculated that miR-330-3p promoted cell invasion and induced EMT may partially via targeting GRIA3 and then upregulating TGF-β1 in NSCLC cells.

## DISCUSSION

BM is the leading cause of death among patients with NSCLC. There is thus an urgent need to identify biomarkers of metastatic potential in this disease to elucidate the underlying mechanisms. Identifying patients at higher risk of developing BM will facilitate prophylactic measures to lower morbidity and mortality. To date, however, no specific markers have been found to identify NSCLC patients susceptible to BM. In two previous studies involving over 300 stage-III/IV Chinese patients, univariate analysis showed that female gender, age < 60 years, and non-squamous cell carcinoma were the risk factors for BM [27, 28]. Whether EGFR mutation is a reliable biomarker for BM in NSCLC remains controversial: two studies suggest EGFR mutation status is linked to BM [29, 30], whereas another study found similar rates of BM in NSCLC patients with wild-type or mutant EGFR [31]. In patients with mutant EGFR, the incidence of BM is reportedly higher in those with mutations at exon 19 than at other sites [27, 28]. The present study confirmed several previously reported risk factors for BM: the female gender, age under 60 years, adenocarcinoma, N2 or N3 lymph node metastases, and mutation at *EGFR* 19 exon.

A critical finding in the present study is that serum and tissue miR-330-3p levels are higher in NSCLC patients with BM than in those without BM. A univariate analysis performed to assess the association between the expression of six miRNAs (miR-325, miR-326, miR-328, miR-330-3p, miR-370, and miR-500-3p) and BM suggested that miR-330-3p is a risk factor for BM in NSCLC. Experiments that manipulated miR-330-3p in A549 and HCC827 cells showed that decreasing miR-330-3p expression promoted cell apoptosis, interfered with cell cycle progression, and inhibited proliferation of NSCLC cells *in vitro* and *in vivo.*

Angiogenesis is a hallmark of tumor progression [32]. NSCLC cells release several cytokines, which act to form autocrine and paracrine growth loops involving NSCLC cells and the microenvironments of BM foci [33]. VEGF is one of the major proangiogenic cytokines inducing neo-angiogenesis in NSCLC [34]. In the present study, we found that VEGF secretion and angiogenesis were reduced by miR-330-3p knockdown in NSCLC cells, suggesting that proangiogenic actions of miR-330-3p may contribute to BM.

Through target gene prediction based on bioinformatics and whole genome microarray analyses, we showed that GRIA3 gene is a downstream target of miR-330-3p. As one of the four subunits of the ionotropic glutamate receptor, GRIA3 is mainly expressed in the central nervous system (CNS). Expression of GRIA3 and other glutamate receptors has been associated with the development and progression of gliomas and neuroblastomas [35, 36]. Additionally, GRIA3 is an important mediator of tumor progression in pancreatic cancer *in vitro* and *in vivo* [37]. We also showed that expression of GRIA3 was inhibited at both the mRNA and protein levels by overexpressing miR-330-3p, and silencing miR-330-3p had the opposite effect. Furthermore, overexpression of GRIA3 reversed oncogenic effects induced by miR-330-3p. Experiments in the patient samples showed that serum GRIA3 levels are lower in BM+ NSCLC patients than BM- patients. Low GRIA3 expression correlated with poor prognosis in lung adenocarcinoma patients. These findings suggest that miR-330-3p promotes cell proliferation and invasion, at least in part by targeting GRIA3.

We further explored the mechanism underlying miR-330-3p promotion of NSCLC cell migration and invasion. A previous study showed that initiation of EMT in tumor cells is the major cause of lung cancer metastasis [38]. TGF-β1 promotes lung adenocarcinoma invasion and metastasis via a mechanism involving EMT [39]. The crosstalk between miRNAs and TGF-β-mediated EMT and tumor metastasis has been comprehensively reviewed in different cancer types [40]. In the present study, we investigated the influence of miR-330-3p on TGF-β-induced EMT and found that miR-330-3p knockdown in NSCLC cells inhibited TGF-β-induced EMT. We further showed that TGF-β1 could interact with GRIA3. We also observed that TGF-β1 and GRIA3 were co-expressed in lung cancer patient samples in the MEM database. These results suggested that miR-330-3p mediated NSCLC cells migration and EMT via GRIA3-TGFβ1 interaction.

In summary, our study identified risk factors for BM in NSCLC and showed that miR-330-3p overexpression is predictive of future development of BM. Our findings also indicate the importance of miR-330-3p in NSCLC progression and highlight the molecular mechanisms underlying miR-330-3p-mediated NSCLC cell migration, invasion, and EMT. Targeting miR-330-3p may be a novel therapeutic strategy for preventing BM in NSCLC patients.

## METHODS

### Patient samples

Study subjects were 122 patients with histologically confirmed NSCLC (using AJCC criteria) receiving treatment during a period from January 2012 to December 2013. This study was approved by the Institutional Review Board of Huazhong University of Science and Technology, Wuhan, China (No: IORG0003571). Written informed consent was obtained from all subjects. Blood samples were collected prior to any treatment. BM was established by confirmed oncologists based on whole brain CT scan or MRI. Patients without BM upon diagnosis (n = 60) received whole brain CT scan after every two cycles of chemotherapy to identify potential development of BM. Peripheral blood samples (3.5 ml) were harvested from subjects and put into anticoagulant-free tubes. Whenever possible, BM+ NSCLC patients were matched with their BM- NSCLC counterparts in terms of age, gender, histology, and stage at diagnosis. These samples were then centrifuged at 1000 rpm for 10 minutes at room temperature. Serum was transferred into RNA-free EP tubes and stored at -80 °C before RNA extraction. miR-330-3p was also measured in fresh lung tumor tissues (obtained with biopsy).

Tumor EGFR mutation status in exons 18 to 21 was determined by examining DNA extracted from formalin-fixed, paraffin-embedded archival tumor tissues on an amplification refractory mutation system (ARMS).

General data, including demographic information and smoking status, are summarized in Supplementary Table 1.

### Cell lines and reagents

A549 and HCC827 cells were obtained from the Institute of Biochemistry and Cell Biology of the Chinese Academy of Sciences. All cells were cultured in RPMI 1640 (Gibco, Grand Island, NY, USA) medium supplemented with 10% fetal bovine serum (10% FBS), 100 U/ml penicillin and 100 mg/ml streptomycin (Invitrogen, Carlsbad, CA, USA) in humidified air at 37 °C with 5% CO_2_. Human umbilical vein endothelial cells (HUVECs) were established as described [41].

### Quantitative reverse transcription-polymerase chain reaction (qRT-PCR)

Total RNA was extracted from 300 μL of serum using a miRNeasy serum and plasma kit (Invitrogen), as described previously [42]. TaqMan miRNA assays were used to detect and quantify miRNAs expression, and U6 was used as an internal control. According to the median of miRNA expression in the cohort of 122 NSCLC patients, we divided the values above or below the median into high or low miRNA expression group in the univariate analysis and multivariate analysis.

For GRIA3 mRNA determination, cDNA was synthesized from total RNA using PrimeScript RT reagent Kit (Takara, Dalian, China) and used as the template for quantification of GRIA3 levels by using SYBR Green RT-PCR Kit (Takara). GAPDH served as an internal control. All PCR reactions were carried out using StepOne Real-time PCR system (Applied Biosystems, Foster City, CA, USA), and data were analyzed using the comparative 2^-Δ^^ΔCT^ method. Three separate experiments were performed for each clone.

### Plasmids and stable transfection

Genomic sequence (*pre–miR-330-3p* or *hsa-miR-330-3p*) was inserted into the retroviral plasmid pMSCV-puro (Genehem, Shanghai, China). The resulting plasmid (pMSCV-miR-330-3p or pMSCV-hsa-miR-330-3p) was used to transfect cells together with the pIK packaging plasmid in 293T cells using a standard calcium phosphate transfection method [43]. The titer of lentivirus was determined with serial dilution method. Then, A549 and HCC827 cells were seeded into 96-well plates, followed by addition of 1 × 10^8^ TU/ml lentivirus, 5 μg/ml polybrene and Enhanced Infection Solution (Genechem). Cells were incubated in an environment of 5% CO_2_ at 37 °C for 24 h. The medium was refreshed, followed by culturing for additional 48 h. Cells were observed under a fluorescence microscope to evaluate the transfection efficiency.

### Western blotting

Total protein (30-50 μg) lysates were subjected to electrophoresis on 10% or 12% sodium dodecyl sulfate-polyacrylamide gel electrophoresis (SDS-PAGE), and transferred onto PVDF membranes (Millipore, Billerica, MA, USA). After blocking with 5% nonfat milk for 1 h, the membranes were incubated with primary antibodies overnight at 4 °C, followed by incubation with the corresponding secondary antibodies (Invitrogen) for 1 h at room temperature. Immunoreactive bands were visualized using the ECL detection system (Thermo Fischer, Waltham, MA, USA) and signal quantification was normalized to GAPDH. The primary antibody sources are listed in Supplementary Table 5.

### Determination of viability, apoptosis and cell cycle

Cell viability was determined using a standard MTT assay, as described previously [44]. Cells were plated 5000 per well in RPMI-1640 supplemented with 10% FBS in 96-well plates, and cultured overnight. Then cells were incubated with 0.5 mg/mL MTT for 4 h. After incubation, 150 μL crystal dissolving buffer was added and cells were shaken for 10 min. The absorbance was measured at 490 nm using Multimode Plate-Reader (PerkinElmer).

Cells undergoing apoptosis and necrosis were measured using PE Annexin V Apoptosis Detection Kit I (BD Bioscience, San Jose, CA, USA). Cell cycle distribution was determined by propidium iodide staining followed by flow cytometry using a FACScan (Becton Dickinson, Franklin Lakes, NJ, USA) [45].

### Microarray analysis

Total RNA isolated from A549 cells transfected with empty lentivirus and with anti-miR-330-3p lentivirus was labeled with Cy5, and then hybridized to Human Whole Genome OneArray^TM^ (Version 6.1, Phalanx Biotech Group). Results were scanned with an Axon 4000 scanner (Molecular Devices) and analyzed using a Genepix software package (Molecular Devices). Candidate genes were identified with filtering at fold change at > 2.0. The data have been deposited in the NCBI's Gene Expression Omnibus and are accessible through GEO Series accession number GSE121323 (https://www.ncbi.nlm.nih.gov/geo/query/acc.cgi?acc=GSE121323).

### Wound-healing assay

Cells were seeded at a density of 5 × 10^5^ cells in six-well plates and cultured until 90% confluency. The cell layer was scratched with a 200 μl sterile plastic tip, washed with PBS and re-cultured with culture medium containing 1% FBS. Cells migrate into the scratch area as single cells from the confluent sides; the width of the scratch gap was viewed under an inverted microscope (Olympus, Tokyo, Japan) and photographed at 0 h, 24 h and 48 h. Three replicate wells from a six-well plate were used for this experiment.

### Transwell migration and invasion assays

For migration assay, 5 × 10^4^ cells were transferred from serum-free media into the upper chamber of the 8-μm pore size membrane. The invasion assays were conducted using 6 × 10^5^ cells in serum-free media transferred on the upper chamber of an insert coated with Matrigel (BD Biosciences). Medium containing 10% FBS was added to the lower chamber. After 24h of incubation, the cells remaining in the upper membrane were removed with cotton applicators. The cells migrated or invaded through the membrane were stained with methanol and 0.1% crystal violet, photographed and counted under an IX71 inverted microscope (Olympus).

### Tube formation assay

Matrigel (50 μl/well, BD) was added to 96-well plates, and polymerized for 2 h at 37 °C. Cells (2-3 × 10^4^ per well) were added and cultured for 6-8 h in serum-free medium prior to image capture under a microscope at 100 × magnification (Olympus). The tube number of branches (the branching points are parts of the skeleton where three or more tubes converge) and number of loops [a loop is an area of the background enclosed (or almost) by the tubular structure] were counted.

### Angiogenesis assays

Invasiveness and tube formation of HUVECs were measured in the presence of conditioned medium (CM) obtained from A549 or HCC827 under specified treatment conditions, as described previously [46].

### Luciferase reporter assay

Wild-type or mutant 3’-untranslated regions (UTR) of GRIA3 were cloned into the pMIR-REPORT miRNA Expression Reporter Vector (Life Technologies, Pittsford, NY, USA). A549 cells were co-transfected with pre-miR-330-3p or controls and wild-type or mutant 3’-UTR-luc, as well as pRL-TK vector as an internal control for luciferase activity. 48 hours post-transfection, the cells were lysed and luciferase assays were conducted using the dual luciferase assay system (Promega, Madison, WI, USA). Each experiment was performed in triplicate.

### Subcutaneous xenograft experiments

Adult female BALB/C nude mice (4-5 weeks of age; Beijing Huafukang Bioscience Company, Beijing, China) were housed in a specific-pathogen-free environment. A549 or HCC827 cells permanently overexpressing miR-330-3por expressing ananti-miR-330-3p were injected subcutaneously (1 × 10^7^ in 100 μL serum-free RPMI-1640 medium). Tumor growth were measured every 3 days and presented as tumor volume (V) using the formula: V = 0.5 × a × b^2^, where a and b represent the longer and shorter tumor diameters, respectively. Four weeks later, mice were sacrificed for immunohistochemical and immunofluorescence examination. A separate group of 20 mice were used for survival analysis, in which mice were euthanized upon clear signs of prolonged distress, neurological impairment, or > 20% body weight loss.

After tumor formation, mice were tranquilized with ether and placed on a bioluminescence imaging system (Xenogen, Berkeley, CA, USA) to measure GFP fluorescence from tumors [47]. All experiments with mice were approved by the Institutional Animal Care and Use Ethics Committee of Huazhong University of Science and Technology.

### Immunohistochemical and immunofluorescence staining

Immunohistochemistry and immunofluorescence staining were performed as described previously [48, 49]. The primary antibodies included: PCNA, cyclinD1 (Abcam), Bcl-2 (Cell Signaling Technology), caspase-3 (Santa Cruz Biotechnology), CD31 (Abcam), E-cadherin (Proteintech, Chicago, IL, USA), Vimentin (Proteintech), CD44 (Proteintech) and β-catenin (Cell Signaling Technology). The secondary antibody for immunofluorescence staining was Alexa Fluor 594 goat anti-mouse Immunoglobulin G (IgG, Beyotime). The sections were examined under a fluorescence microscope (Olympus).

### Brain metastatic xenografts

Adult female BALB/C nude mice (6-7 weeks of age; Beijing Huafukang Bioscience Company were fixed on a stereotactic apparatus. A 2- to 3- mm incision was made along the cranial midline. Cells (3 × 10^5^ in 10-μL PBS) were injected into the brain unilaterally at the following coordinates: L: 2.0 mm, AP: -0.5 mm, D: 3.5 mm using a microsyringe [50]. Mice were examined using a 7T Magnetic Resonance Imaging (MRI). T2-weighted MRI images were used for data analyses.

### Gelatin zymography

MMP Zymography assay kit (for MMP2 and MMP9) (Applygen Technologie, China) was used to detect the activity of MMP2 and MMP9. Protein extracts were mixed with an equal volume of 2 × SDS-PAGE non-reducing buffer, and electrophoresed on 10% SDS polyacrylamide gels containing 10 mg/ml of gelatin. Gels were then washed twice for 30 min in buffer A at room temperature, and incubated for 48 hours at 37 °C in incubation buffer B. Gels were then stained for 2 hour with 0.25% Coomassie brilliant blue and then destained in destaining buffer (10% acetic acid and 20% methanol) for 60 min.

### Statistical analysis

Data are presented as mean ± standard deviation (SD). The log-rank test (based on K-M analysis) and Cox proportional hazards regression were used to analyze the association of clinical variables and miRNAs with BM. The levels of microRNAs were analyzed using the Mann-Whitney U rank sum test. Differences between 2 groups were determined by unpaired 2-tailed Student’s *t* test. Differences between multiple groups were determined by one-way ANOVA with post-hoc Tukey HSD test. All statistical tests were two-sided. *P* < 0.05 was considered statistically significant.

### Ethics approval

Investigation has been conducted in accordance with the ethical standards and according to the Declaration of Helsinki and according to national and international guidelines and has been approved by the Institutional Review Board of Huazhong University of Science and Technology, Wuhan, China.

## Supplementary Material

Supplementary Figures

Supplementary Tables
