# Supplementary material for: MicroRNA-330-3p promotes brain metastasis and epithelial-mesenchymal transition via GRIA3 in non-small cell lung cancer
Source: Aging (Albany NY). 2019 Sep 8;11(17):6734–61. doi: 10.18632/aging.102201 (PMC6756898; doi:10.18632/aging.102201)
Supplement: Supplementary Figures [file aging-11-102201-s002.pdf]

SUPPLEMENTARY FIGURES

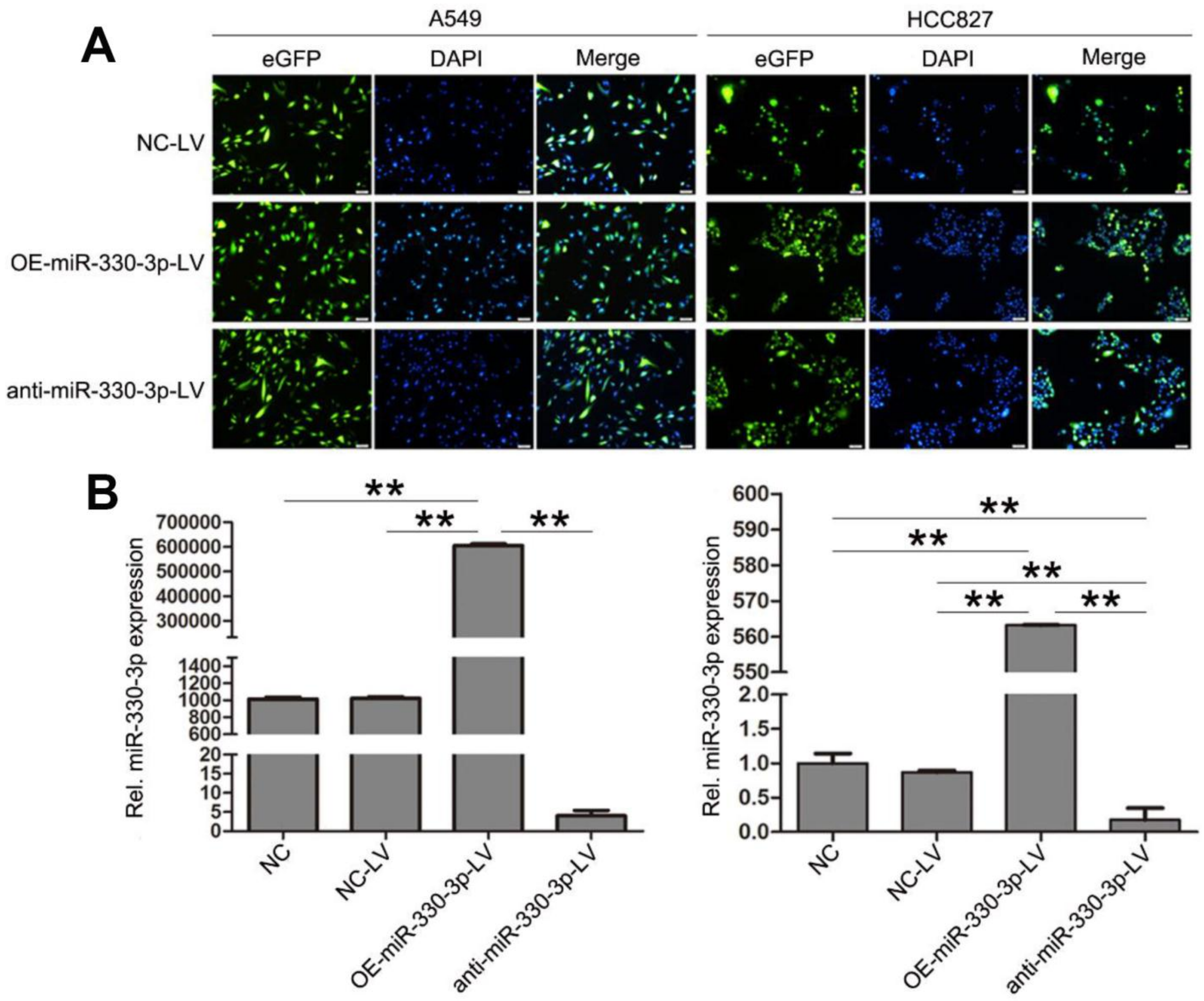

Supplementary Figure 1. Stable transfected A549 and HCC827 cells. (**A**) Green fluorescent protein (GFP) expression after A549 and HCC827 cells transfection with lentivirus. Transfection efficiency was assessed by immunofluorescence staining (original magnification ×100). (**B**) After stable transfection, RNA was extracted and the miR-330-3p level was determined by qRT-PCR analysis. The amount of miR-330-3p was normalized to U6. **P* < 0.05, ***P* < 0.01. NC: Cells not subjected to viral transfection; NC-LV: cells transfected with empty lentivirus; OE-miR-330-3p-LV: cells transfected with lentivirus over-expressing miR-330-3p; anti-miR-330-3p-LV: cells transfected with anti-miR-330-3p lentivirus.

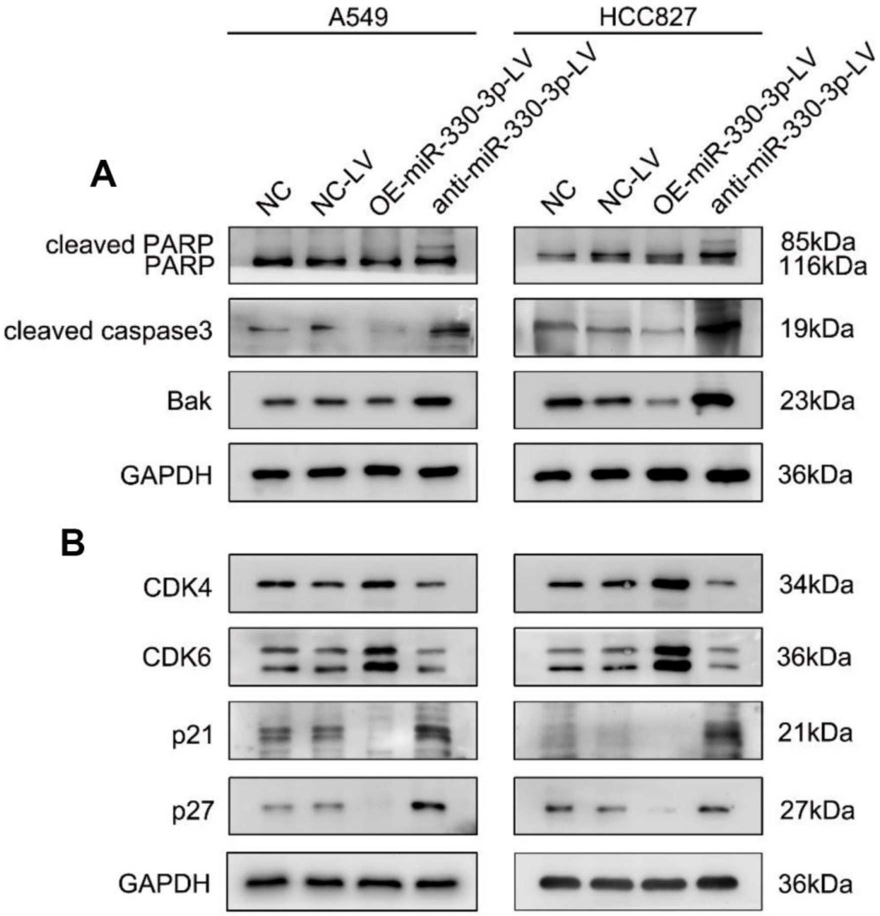

Supplementary Figure 2. MiR-330-3p regulated cell apoptosis and cell cycle of NSCLC cells. (**A**, **B**) Western blotting analysis evaluated the levels of cell apoptosis related proteins (**A**) and cell cycle regulatory proteins (**B**) in A549 and HCC827 cells.

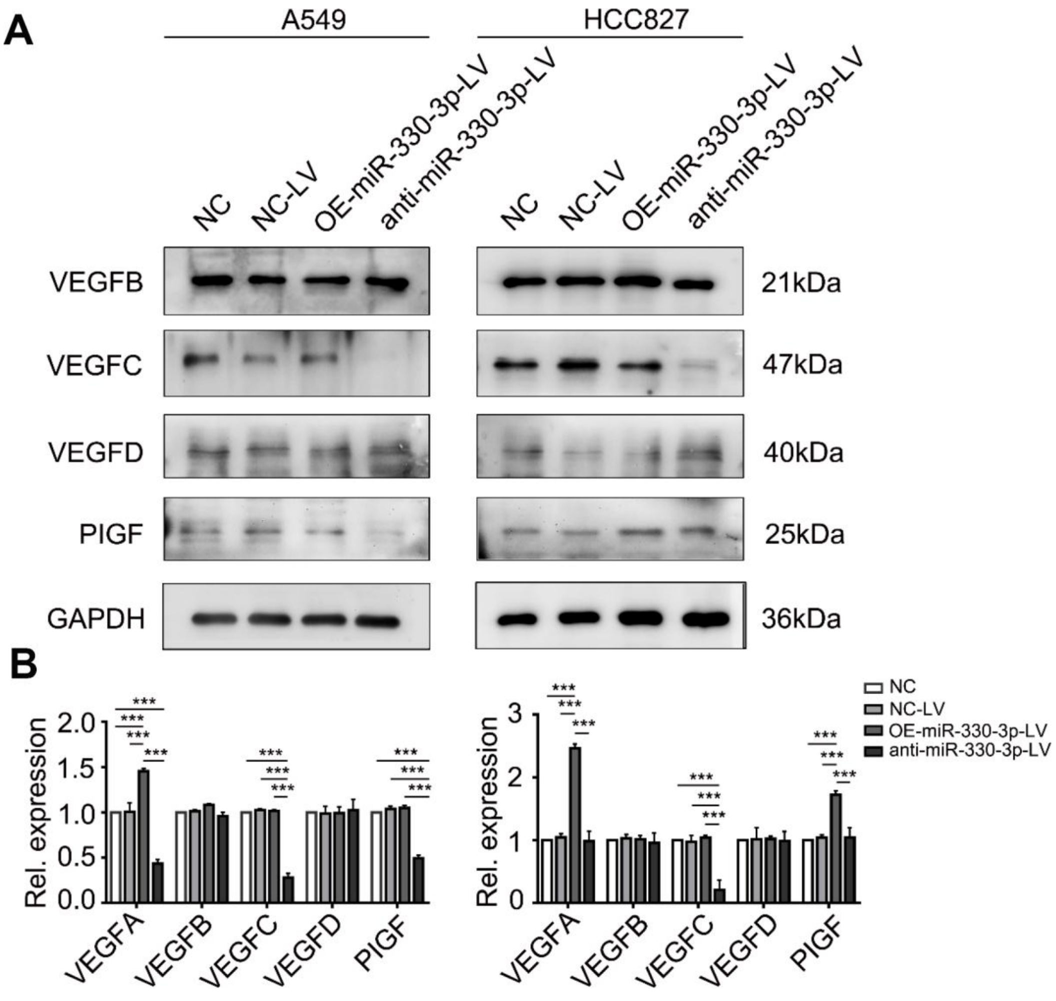

Supplementary Figure 3. MiR-330-3p promoted the expression of VEGF family of HUVEC cells co-cultured with A549 and HCC827 cells. (**A**, **B**) Over-expressing miR-330-3p elevated the level of VEGFA and PIGF expression, knockdown of miR-330-3p inhibited the expression of VEGFA and VEGFC by western blotting (**A**) and qRT-PCR (**B**). ***$P < 0.001$.

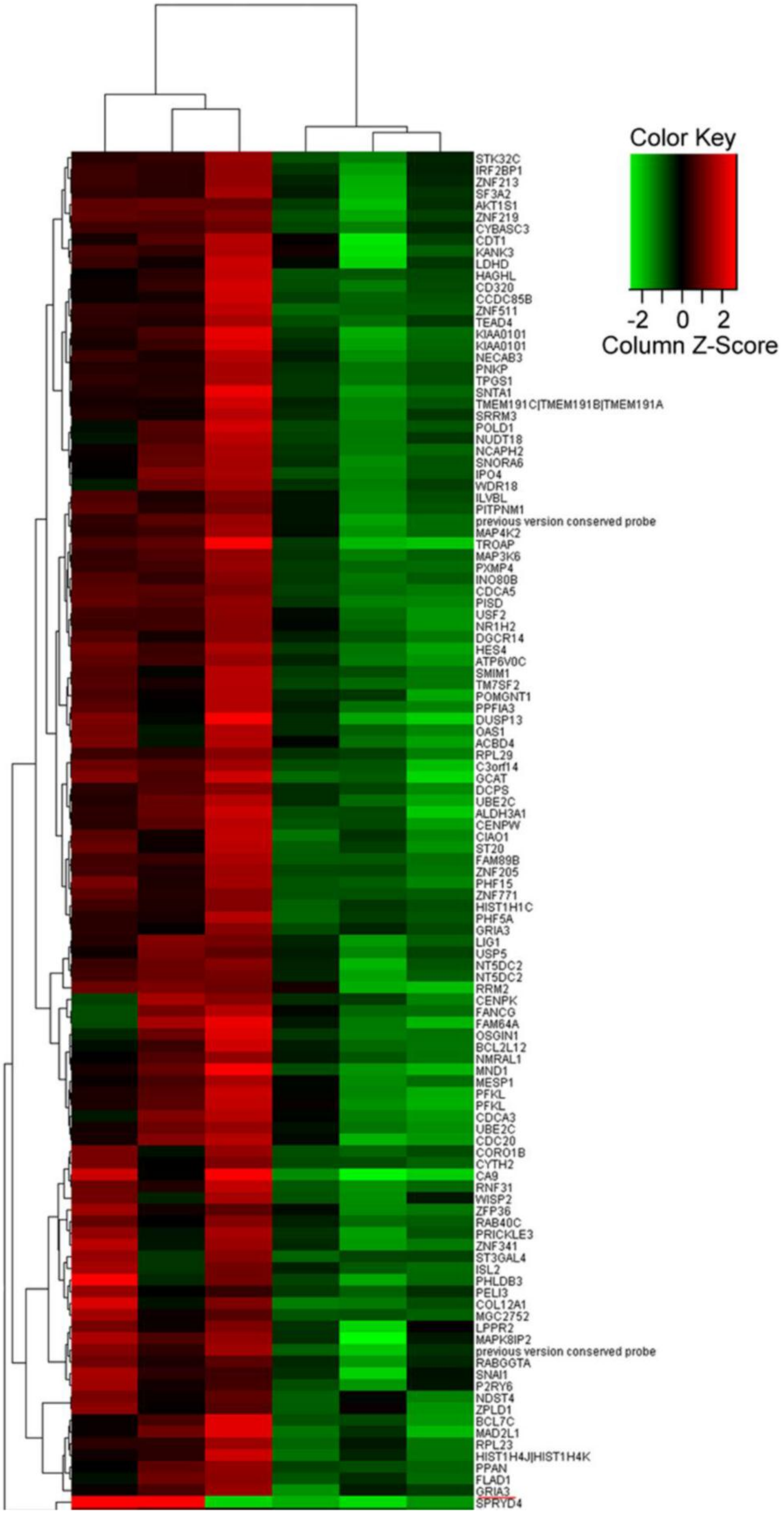

Supplementary Figure 4. Hierarchical clustering of differentially expressed genes in anti-miR-330-3p and NC-LV-treated A549 cells. Genes that were affected 42-fold with *P*-values below 0.05 were selected, classified and clustered.

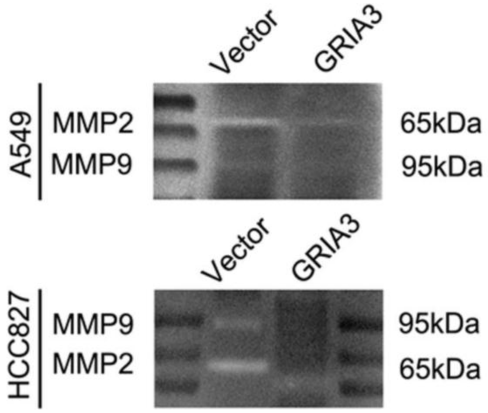

Supplementary Figure 5. Gelatin zymography assay was performed to detect the activity of MMP2 and MMP9 in A549 and HCC827 cells overexpressing GRIA3.

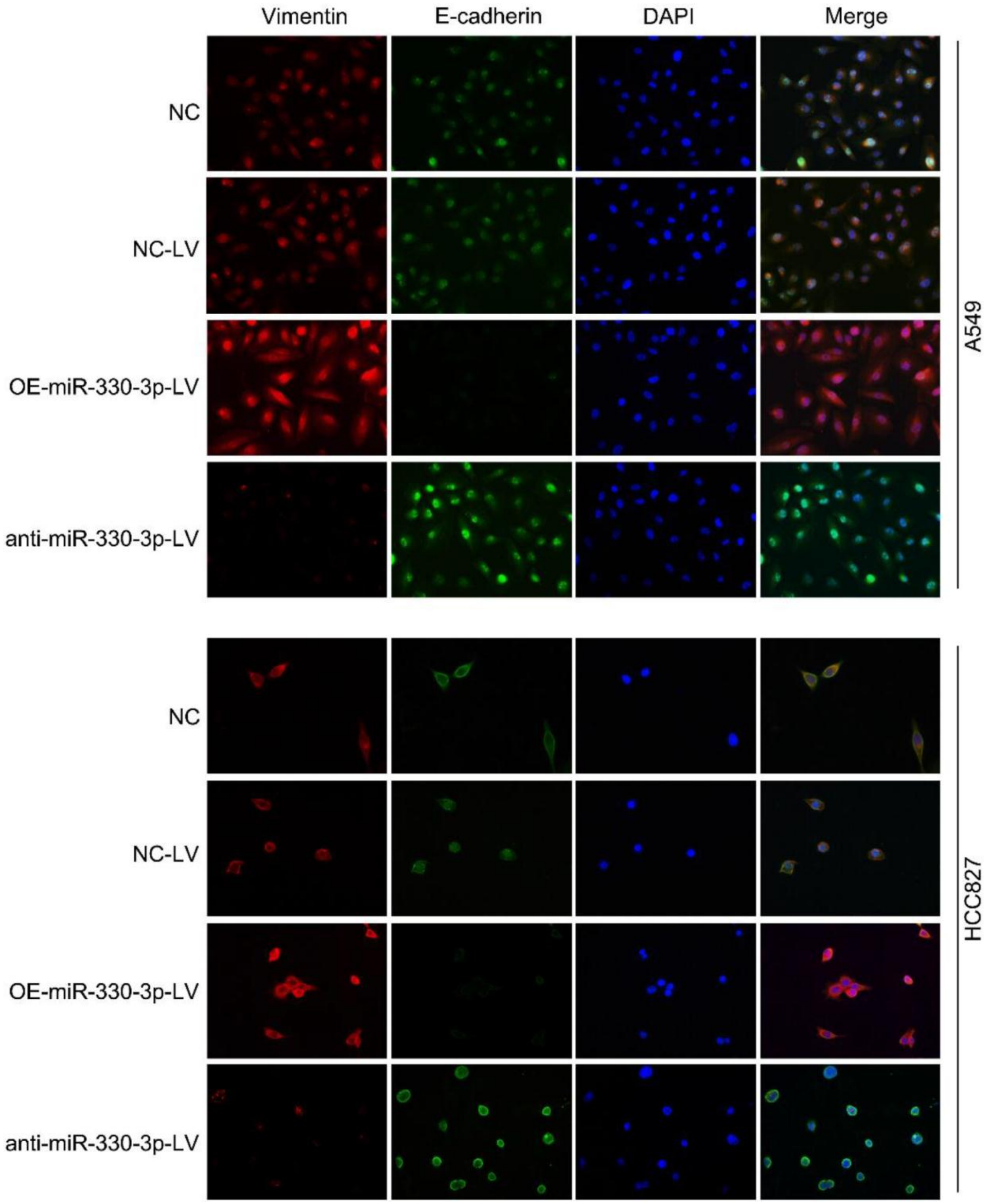

Supplementary Figure 6. Immunofluorescence staining detected the Vimentin and E-cadherin expression in A549 and HCC827 cells.

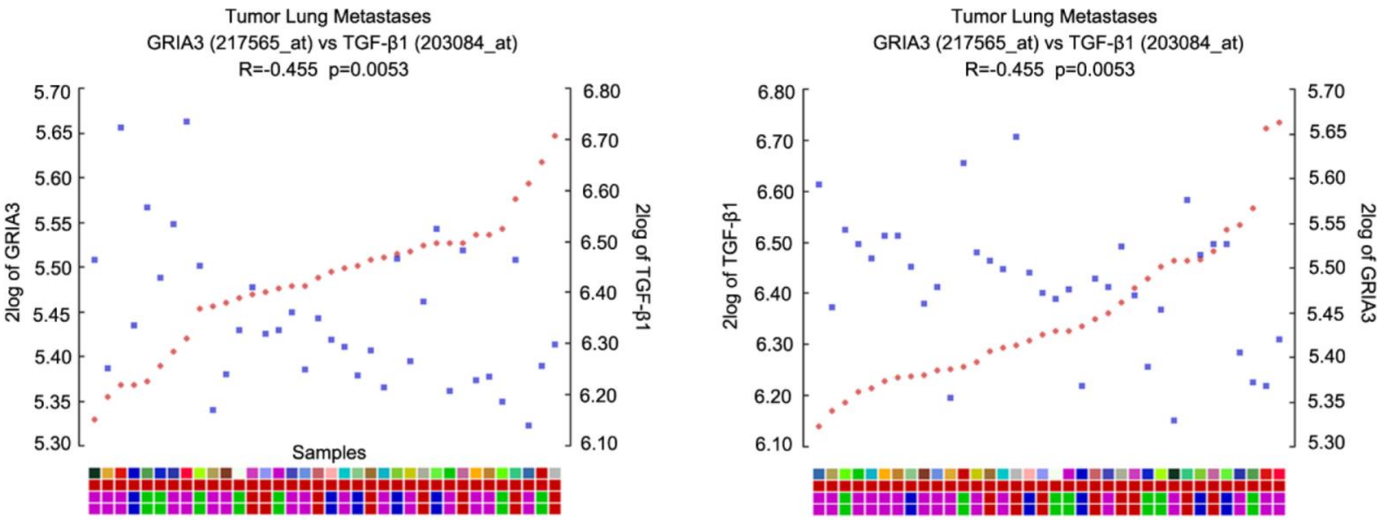

Supplementary Figure 7. GRIA3 expression was negatively associated with TGF-β1 in clinical NSCLC metastases specimens (R = -0.455, P = 0.0053). Gene correlation analysis was based on the TCGA NSCLC dataset and was analyzed via the R2: Genomics Analysis and Visualization Platform.
