# Supplementary material for: MicroRNA-330-3p promotes brain metastasis and epithelial-mesenchymal transition via GRIA3 in non-small cell lung cancer
Source: Aging (Albany NY). 2019 Sep 8;11(17):6734–61. doi: 10.18632/aging.102201 (PMC6756898; doi:10.18632/aging.102201)
Supplement: Supplementary Tables [file aging-11-102201-s001.pdf]

SUPPLEMENTARY TABLES

Supplementary Table 1. Clinical characteristics of 62 NSCLC patients with brain metastasis and 60 patients without brain metastasis.

Characteristic	NSCLC with BM total 62	NSCLC without BM total 60
Age (years), median (range)	58 (50-65)	60 (55-67)
Gender		
Male	13 (21.0%)	37 (61.7%)
Female	49 (79.0%)	23 (38.3%)
Tumor histology		
Adenocarcinoma	54 (87.1%)	41 (68.3%)
Squamous cell carcinoma	8 (12.9%)	10 (16.7%)
Carcinosarcoma	-	2 (3.3%)
Large cell carcinoma	-	1 (1.7%)
Neuroendocrice carcinoma	-	6 (10.0%)
Disease stage at diagnose		
I	-	6 (10.0%)
II	-	12 (20.0%)
III	-	11 (18.3%)
IV	62 (100%)	31 (51.7%)
Smoking history		
Smoker	18 (29.0%)	8 (13.3%)
Former smoker	6 (9.7%)	22 (36.7%)
Never	38 (61.3%)	30 (50.0%)
Lymph node metastatic status		
N0	4 (6.5%)	9 (15.0%)
N1	2 (3.2%)	7 (11.7%)
N2	22 (35.5%)	17 (28.3%)
N3	34 (54.8%)	27 (45.0%)
EGFR mutation status		
Mutant	42 (67.7%)	32 (53.3%)
Wild-type	20 (32.3%)	28 (46.7%)

Supplementary Table 2. Univariate analysis and Multivariate analysis of associations between clinical features and brain metastasis.

Variable	Univariate analysis			Mutivariate analysis		
	OR	95%CI	*P*	OR	95%CI	*P*
**Gender**<br>Female *vs* Male	2.84	1.55~5.21	0.001	3.15	1.49~6.63	0.003
Age (year)<br>≥60 *vs* <60	3.45	0.16~0.52	< 0.001	4.54	0.11~0.46	<0.001
**Smoking status**						
Former smoker *vs* Never	0.75	0.35~1.6	0.458			
Smoker *vs* Never	1.38	0.62~3.08	0.424			
**Tumor histology**						
Adnocarcinoma *vs* Other types	4.17	0.1~0.53	0.001	3.57	0.1~0.75	0.012
**EGFR status**						
19 exon mutated *vs* Wildtype	3.28	1.13~9.56	0.03	3.76	1.00~14.12	0.05
21 exon mutated *vs* Wild type	1.76	0.6~5.18	0.307			
**Lymph node metastatic status**						
N1 *vs* N0	5	0.76~32.93	0.094	6.14	0.8~47.19	0.081
N2 *vs* N0	7.88	1.69~36.82	0.009	8.23	1.57~43.01	0.013
N3 *vs* N0	19.06	4.19~86.74	< 0.001	29.38	5.68~152.04	<0.001
**Disease stage at diagnose**						
Stage III *vs* Stage I, II	1.89	0.01~2.56	0.177			
Stage IV *vs* Stage I, II	2.12	0.13~34.71	0.599			
**PCR**						
high expression *vs* low expression						
miR-370	1.167	0.302~4.512	0.823			
miR-325	0.435	0.147~1.289	0.133			
miR-326	0.318	0.086~1.182	0.087	0.523	0.099~1.231	0.032
miR-330-3p	0.159	0.048~0.531	0.003	0.174	0.079~0.679	0.001
miR-350	0.09	0.022~0.365	0.001			
miR-328	0.067	0.016~0.273	<0.001	0.073	0.023~0.281	0.002

Supplementary Table 3. Univariate analysis of miRNA expression and brain metastasis.

miRNA	Sig.	95% CI	
		Lower	Upper
miR-325	0.120	2.323	5.793
miR-328	0.048	1.521	3.011
miR-330-3p	0.023	2.630	16.20
miR-500-3p	0.087	1.218	2.831
miR-326	0.774	1.718	2.956
miR-370	0.384	1.347	2.728

Sig., *p*-value; CI, confidence interval.

Supplementary Table 4. The sequence of predicted targets of miR-330-3p.

Predicted targets	Paring of miRNA/targets
Position 223-229 of GRIA3 3′UTR	5′...UCAUUGAAAUCUUUUUGCUUUGC...
miR-330-3p	3′ AGAGACGUCCGGCACACGAAACG
Position 113-120 of SOSTDC1 3′UTR	5′...AAAGUAUAUGCUUUCUGCUUUGA...
miR-330-3p	3′ AGAGACGUCCGGCACACGAAACG
Position 1484-1490 of BMI-1 3′UTR	5′...GAAGCACAAUUCUAUUGCUUUGU...
miR-330-3p	3′ AGAGACGUCCGGCACACGAAACG
Position 846-853 of AGTR2 3′UTR	5′.. ACUUUUUAGAUGUGCUGCUUUGA...
miR-330-3p	3′ AGAGACGUCCGGCACACGAAACG

GRIA3, ionotropic glutamate receptors, AMPA3; SOSTDC1, sclerostin domain containing 1; BMI-1, B lymphoma Mo-MLV insertion region 1; AGTR2, Angiotensin Type-2 Receptor.

Supplementary Table 5. The primary antibodies for western blotting.

Antibodies	Accession
cyclin D1	ab6152, Abcam, Cambridge, MA, USA
Bcl-2	15071, Cell Signaling Technology
Bax	ab77566, Abcam
VEGFA	ab46154, Abcam
GRIA3	ab52896, Abcam
GAPDH	G9545, Sigma, St Louis, MO, USA
The EMT Antibody Sampler Kit	9782, Cell Signaling Technology
PARP	13371-1-AP, Proteintech, Chicago, IL, USA
Caspase3	19677-1-AP, Proteintech
p21	10355-1-AP, Proteintech
p27	25614-1-AP, Proteintech
CDK4	11026-1-AP, Proteintech
CDK6	14052-1-AP, Proteintech
VEGFC	22601-1-AP, Proteintech
Twist	25465-1-AP, Proteintech
Bak	A0204, ABclonal Biotech, Cambridge, MA
PIGF	A13617, ABclonal Biotech
VEGFB	A12689, ABclonal Biotech
VEGFD	A13617, ABclonal Biotech
p-β-catenin	4176, Cell Signaling Technology
